# Current indications and surgical strategies for myocardial revascularization in patients with left ventricular dysfunction: a scoping review

**DOI:** 10.1186/s13019-024-02844-2

**Published:** 2024-07-27

**Authors:** Alejandro Moreno-Angarita, Diego Peña, Juan David Lopez-Ponce de León, Mayra Estacio, Lidy Paola Vila, Maria Isabel Muñoz, Eduardo Cadavid-Alvear

**Affiliations:** 1 Fundación Valle del Lili - Departamento de Cirugía - Servicio de Cirugía Cardiovascular, Carrera 98 No. 18-49, Cali, Valle del Cauca 760032 Colombia; 2 Fundación Valle del Lili - Centro de Investigaciones Clínicas, Cali, Colombia; 3 Fundación Valle del Lili - Departamento de Medicina Interna, Cali, Colombia; 4 Fundación Valle del Lili - Departamento de Cardiología, Cali, Colombia; 5 Universidad Icesi - Departamento de Ciencias de la Salud, Cali, Colombia

**Keywords:** Left ventricular dysfunction, Surgical revascularization strategies, Grafting options, Heart failure

## Abstract

**Background:**

Ischemic cardiomyopathy (ICM) accounts for more than 60% of congestive heart failure cases and is associated with high morbidity and mortality rates. Myocardial revascularization in patients with left ventricular dysfunction (LVD) and a left ventricular ejection fraction (LVEF) ≤35% aims to improve survival and quality of life and reduce complications associated with heart failure and coronary artery disease. The majority of randomized clinical trials have consistently excluded those patients, resulting in evidence primarily derived from observational studies.

**Main body:**

We performed a scoping review using the Arksey and O'Malley methodology in five stages: 1) formulating the research question; 2) locating relevant studies; 3) choosing studies; 4) organizing and extracting data; and 5) compiling, summarizing, and presenting the findings. This literature review covers primary studies and systematic reviews focusing on surgical revascularization strategies in adult patients with ischemic left ventricular dysfunction (LVD) and a left ventricular ejection fraction (LVEF) of 35% or lower. Through an extensive search of Medline and the Cochrane Library, a systematic review was conducted to address three questions regarding myocardial revascularization in these patients. These questions outline the current knowledge on this topic, current surgical strategies (off-pump vs. on-pump), and graft options (including hybrid techniques) utilized for revascularization.

Three independent reviewers (MAE, DP, and AM) applied the inclusion criteria to all the included studies, obtaining the full texts of the most relevant studies. The reviewers subsequently assessed these articles to make the final decision on their inclusion in the review. Out of the initial 385 references, 156 were chosen for a detailed review. After examining the full articles were examined, 134 were found suitable for scoping review.

**Conclusion:**

The literature notes the scarcity of surgical revascularization in LVD patients in randomized studies, with observational data supporting coronary revascularization's benefits. ONCABG is recommended for multivessel disease in LVD with LVEF < 35%, while OPCAB is proposed for older, high-risk patients. Strategies like internal thoracic artery skeletonization harvesting and postoperative glycemic control mitigate risks with BITA in uncontrolled diabetes. Total arterial revascularization maximizes long-term survival, and hybrid revascularization offers advantages like shorter hospital stays and reduced costs for significant LAD lesions.

## Background

Ischemic cardiomyopathy (ICM) is defined as significantly impaired left ventricular function, specifically a left ventricular ejection fraction (LVEF) ≤40%, resulting from coronary artery disease (CAD)[1]. Nevertheless, there is no consensus about a universal definition of this disease. In the following review, we will concentrate on patients with an ICM and LVEF ≤35%. This condition accounts for more than 60% of congestive heart failure cases and is linked to high morbidity and mortality, carrying a substantial risk of major cardiovascular events [2–4].

In patients with ICM, the treatment goals are prolonging survival, enhancing quality of life, and reducing the risk of both cardiac and noncardiac complications. Left ventricular dysfunction (LVD) in coronary disease patients is not necessarily irreversible. The underlying principle justifying the presumed benefit of revascularization is that augmenting blood supply to ischemic 'hibernating' myocardial segments ameliorates symptoms and regional ischemia and improves overall LV function and, consequently, clinical outcomes [3–5]. Patients with ICM have been systematically excluded from the majority of randomized controlled trials (RCTs) concerning the management of chronic coronary syndrome. Hence, there is uncertainty regarding the applicability of published results to this patient population, and the evidence stems from observational studies [6]. The 2021 ACC/AHA guidelines [7] recommend surgical revascularization for patients with an ICM and LVEF <35% to improve survival [8, 9]. However, there are no specific recommendations regarding the optimal revascularization strategy for these patients. On the other hand the ESC/EACTS Guidelines [10] recommend surgical revascularization as the first line of treatment in this specific population when there is an acceptable surgical risk [8, 11–13] (see Table 1).

**Table 1:** Overview of existing guidelines and supporting evidence of myocardial revascularization in patients with LVEF of 35% or less

	**AHA Guidelines **[7]	**ESC/EACTS Guidelines **[10]	**Randomized controlled trials**
**Left ventricular dysfunction**			
**Coronary artery bypass graft (CABG)**	Class I: LVEF <35% without significant left main coronary artery disease.	**LVEF <35%** Class I: First-line revascularization method in multivessel coronary artery disease (CAD) with acceptable surgical risk [8, 11–13].	**STICH**: 30 days mortality 5.1% [14]. **STICHES:** Significant survival benefit at 10 years of follow up [8].
**Percutaneous coronary intervention (PCI)**	Insufficient data.	Class IIa: Single or two-vessel CAD when complete revascularization is achievable. Class IIa: Multivessel CAD, as per evaluation by the Heart Team considering coronary anatomy, expectation of complete revascularization, diabetic status, and comorbidities.	

Multiple studies [15–29] support that the LVEF significantly improves (e.g., ≥5%) after revascularization in up to 60% of patients, reported within a range of 38% [18] to 88% [19]. This effect has been noted in patients exhibiting evidence of hibernating myocardium, contrary to those without hibernation [30], which has been a debated topic.

The primary objective of this review is to condense the current literature and disseminate research findings on variations in surgical techniques and graft options for myocardial revascularization in patients with LVD based on the latest evidence.

## Methods

We conducted a scoping review following the Arksey and O’Malley methodology [24], encompassing the following five stages: 1) defining the research question; 2) identifying pertinent studies; 3) selecting studies; 4) organizing and extracting data; and 5) compiling, summarizing, and presenting the findings.

This scoping review serves as a comprehensive overview of an intricate area or one that has not been thoroughly reviewed previously, aiming to include all relevant literature regardless of study design [31]. We used the following keywords to carry out this research: “Ischemic cardiomyopathy”, “Coronary revascularization”, “Surgical revascularization”, “Left ventricular dysfunction”, “Revascularization strategies”, “Off pump”, “Hybrid revascularization”, “Arterial grafts”, “Heart failure” and “Cardiogenic shock”.

The main objective is to summarize and disseminate research findings on current knowledge, surgical techniques and graft options for myocardial revascularization in patients with left ventricular dysfunction and the current evidence in the literature. A secondary objective is patient selection for each surgical technique and the frequency of using a second arterial graft, total arterial revascularization or hybrid technique.

Aligned with these objectives, the following research questions were developed: What is the existing knowledge regarding surgical techniques for coronary revascularization in patients with left ventricular dysfunction? What revascularization strategy (on pump vs. off pump) should be used in LVD? What graft options, including hybrid techniques, have better outcomes for revascularizing these patients?

To ensure the selection of relevant literature, specific inclusion and exclusion criteria were formulated for all the retrieved data.

The inclusion criterion was:

- Studies involving patients with ischemic left ventricular dysfunction (LVEF ≤35%).
- Studies concerning revascularization strategies in patients with ischemic left ventricular dysfunction.
- All the original studies, irrespective of study design, relevant systematic reviews and published American and European consensus guidelines.

The exclusion criterion was:

- Studies focusing on patients with coronary artery disease but without left ventricular dysfunction.
- Studies published in languages other than English.
- Animal studies.
- Opinion pieces, viewpoints, letters to the editor, conceptual frameworks, and conference abstracts.

Eligible studies were identified through searching the PubMed (MEDLINE database) bibliographical database and the Cochrane Library. Additionally, the reference lists of the included studies were scrutinized, relevant organizations were reviewed, and key journals were manually searched to identify further pertinent publications that might not have been captured in the initial search.

**Fig. 1:**
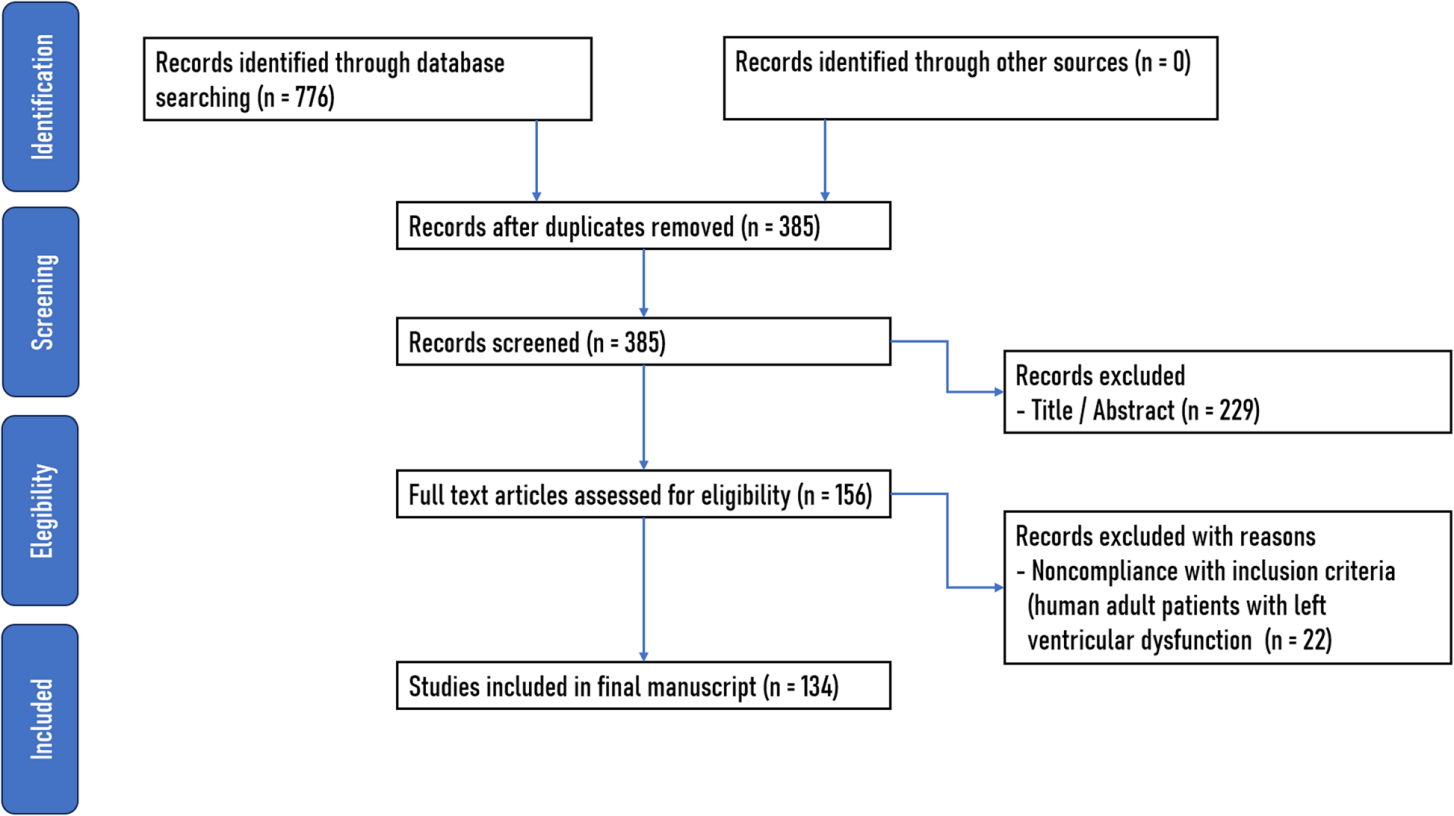
Search algorithm

The application of the inclusion criteria to all the included studies was carried out independently by three reviewers (MAE, DP, and AM). Full articles were acquired for studies appearing most relevant to the research question. Subsequently, the reviewers evaluated the full articles to make the final decision on their inclusion in the review. In instances of potential discrepancies in articles inclusion, each reviewer independently assessed the eligibility criteria. Subsequently, consensus was sought through deliberation and referencing the established criteria. The reviewing team consisted of three members, and this decision was deliberate in order to maintain an odd vote (Fig. 1).

## Main text

From the initial pool of 358 references, we narrowed the sample to 156 for a thorough review. After examining the full articles, 134 were determined to be suitable for inclusion in the scoping review.

## Existing knowledge of myocardial revascularization in patients with LVD 35% or less

However, the optimal revascularization strategy for patients with CAD and severe LV dysfunction remains unclear [13]. European guidelines recommend Class I CABG for multivessel or left main disease, in the presence of angina or heart failure, with acceptable surgical risk. PCI is recommended as a Class IIa indication in patients with one or two-vessel disease and could be considered as a Class IIa indication in patients with three-vessel disease with a low SYNTAX score, considering the patient's expectation of complete revascularization, diabetic status, and comorbidities [10]. AHA guidelines consider CABG for patients with moderate to severe LV dysfunction (35-50%) (Class IIa) and could be considered for severe LV dysfunction (LVEF <35%) (Class IIb) with significant left main disease. PCI is preferred as an alternative to CABG in selected stable patients with significant left main coronary disease, favorable anatomical conditions, or clinical characteristics predicting a significantly greater risk of adverse surgical outcomes [32].

Regarding evidence from clinical trials, the STICH trial [14] randomized 1,012 patients with ICM (LVEF <35%) to CABG plus optimal medical therapy (OMT) or OMT alone. At a median follow-up of 56 months, no significant differences were observed between the two arms in the primary outcome, defined as the rate of death from any cause. However, patients in the CABG group exhibited lower rates of cardiovascular death and death from any cause or hospitalization due to cardiovascular causes. In a subsequent analysis with a median follow-up of 9.8 years (STICH Extended) [8] a 16% reduction in all-cause mortality was found in the CABG group compared to the OMT group (*P* = 0.02). Subgroup analyses indicated that patients with 3-vessel CAD (*P* = 0.04) or severely remodeled LV (end-systolic LV volume index >78 mL/m2 or EF <27%: *P*=0.03) appeared to derive the maximum benefit from revascularization.

Nevertheless, a critical examination of the methodological limitations of the STICH trial is important. A considerable proportion of the study cohort exhibited either the absence of angina symptoms or a Canadian Cardiovascular Society (CCS) Angina Score of 1, a subjective parameter for ischemia assessment. Objective evaluations of ischemia, such as myocardial scintigraphy or stress echocardiography, were not provided. Consequently, the trial’s ability to differentiate between patients exhibiting fibrotic myocardium and those with ischemic myocardium, thereby identifying candidates likely to benefit from revascularization, is questionable. Additionally, a STICH substudy with only 399 patients who underwent ischemic testing revealed no mortality benefit associated with revascularization, irrespective of the presence of ischemia [33]. The inclusion of patients without regard to myocardial viability, despite the absence of evidence supporting the clinical efficacy of viability-guided revascularization, constitutes another notable limitation [25]. Additionally, the substantial crossover rate of 17% potentially introduces bias, potentially underestimating the trial’s results.

Myocardial viability testing can be performed with multiple imaging diagnostic techniques. Dobutamine Stress Echocardiography (DSE) assesses contractile reserve. Dobutamine can be used in low doses (5-10 μg x kg x min) or high doses (40 μg x kg x min) in order to evaluate the contractile reserve and showing promising predictive value for recovery post-revascularization [34], especially with a biphasic response [35]. Another alternative is the Single Photon Emission Computed Tomography (SPECT), which along with the intake of radiotracers in myocardial cells offers valuable information on myocardial perfusion and viability [34, 36–39]. Positron Emission Tomography (PET) with the help of radiotracers bonded to glucose molecules provides superior spatial resolution. Distinguishing between irreversible injured and viable regions [40, 41], relies on the perfusion and metabolism of myocardial cells, but it requires meticulous glucose control for accurate assessment [34]. Another alternative is Cardiovascular Magnetic Resonance (CMR), that utilizing gadolinium chelated contrast agents, allows the precise detection of perfusion defects, microvascular obstruction and the transmurality of scar tissue with the most resolution [25, 34, 42–46]. This information aids to predict the functional recovery post-revascularization. This technique can also be performed along with dobutamine for stress testing [25, 47].

Overall The STICH trial has been utilized by cardiologists and surgeons for over a decade to aid in decision-making for patients with LVD, however it is important to know its limitations. Nowadays, the above described methods to demonstrate ischemia and viability enable the heart team, especially the surgeon, to make judicious decisions for these patients who may benefit from surgical revascularization.

In the SYNTAX trial [48], at the 5-year follow-up, a more pronounced advantage was evident in complex lesions of 3 vessels or left main disease with CABG, and at 10 years of follow-up, the advantage persisted in 3-vessel disease [49]. However, the patients with ICM and an initial LVEF ≤30% constituted a minority within the CABG cohort, accounting for only 2.5% compared to a mere 1.3% in the PCI group.

The HEART trial [50] was originally intended to enroll 800 patients with ICM and severely reduced LVEF (<35%) with residual myocardial viability according to conventional imaging, such as stress echocardiography with dobutamine, angiocardiography, and positron emission tomography (PET). Patients were assigned to OMT or invasive management of revascularization via angioplasty and/or CABG. Only 138 patients were ultimately randomized, and no significant differences were found between patients assigned to one of the management strategies at the 5-year follow-up.

A meta-analysis comparing different revascularization methods (PCI vs. CABG vs. OMT) in patients with coronary artery disease and an LVEF ≤40% demonstrated a significant reduction in mortality with revascularization strategies versus OMT. Moreover, among the revascularization strategies, CABG appears more favorable than PCI [13]. This is attributed to higher survival rates and lower rates of repeat revascularization or reinfarction post-revascularization, albeit with a higher risk of stroke with a minimum follow-up of at least 12 months. However, this does not specify the surgical revascularization technique under which this benefit is observed.

Available evidence supports CABG over PCI in patients with CAD and severely depressed LV systolic function, although the benefit of CABG over OMT seems to become significant only later during long-term follow-up [6]. A limitation of these trials is that medical therapy did not include modern medications, such as angiotensin receptor/neprilysin inhibitors or sodium-glucose cotransporter 2 inhibitors, which have demonstrated a greater impact on major cardiovascular outcomes.

Patients with left ventricular dysfunction pose the greatest challenges and complexities in coronary surgery, with risk scores indicating a high prediction of mortality. This is due to the high likelihood of complications, which increase the probability of early mortality. However, the clinical characteristics that render these patients complex also identify them as the population that could benefit the most from CABG [51]. Additionally, risk scores are not specifically tailored for patients with severe left ventricular dysfunction, hence, their prediction may not accurately reflect the real risk [52].

Most of the evidence supporting myocardial revascularization in ischemic cardiomyopathy (ICM) originates from observational studies, with conflicting data from a limited number of RCTs (see Table 2). Based on this information, guidelines recommend CABG as the preferred revascularization strategy without specifying which technique is most beneficial for these patients.

**Table 2:** Evidence supporting myocardial revascularization in ischemic cardiomyopathy

Study Title / First Author	Year of Publication	Sample Size / Follow Up	Study Objective	Study Main Conclusion	Low Ejection Fraction (n%)	Mortality
S Yusuf	1994	2649 (1324 CABG vs. 1325 MT) Follow up 10 years	Compare the effects of routine CABG with MT on mortality at 5, 7, 10 years among randomized patients.	Routine early CABG improves survival over initial MT. The benefits are pronounced in patients with more extensive coronary disease or ischeaemia and with high or moderate risk.	LVEF <40% (7.2%)	CABG vs. MT: 5 years 10.2% vs. 15.8%, OR 0.61 [CI 95% 0.48 - 0.77] *p* 0.0001; 7 years 15.8% vs. 21.7% OR 0.68 [0.56 - 0.83] *p* <0.001; 10 years 26.4% vs. 30.5% OR 0.83 [0.70 - 0.98] *p* 0.03
STICHES	2016	1212 (610 CABG vs. 602 MT) Follow Up 9.8 years	Evaluate if CABG + optimal MT for CAD, heart failure and LVD would improve survival over MT alone.	Significant benefit of CABG + MT with respect to the rate of death from any cause among patients with ischemic cardiomyopathy.	All population	CABG 359/610 (58.9%) vs. MT 398/602 (66.1%)
STICH	2011	1212 (610 CABG vs. 602 MT) Follow Up 5 years	Evaluate the role of cardiac surgery in treatment of patients with CAD and LVD.	Patients assigned to CABG had lower rates of death from cardiovascular causes and death from any cause or hospitalization for cardiovascular causes.	All population	From cardiovascular causes: MT 201 (33%) vs. CABG 168 (28%) HR 0.81 (0.66 - 1.00) *p* 0.05 From any cause or hospitalization for cardiovascular causes MT 411 (68%) vs. CABG (351 (58%) HR 0.74 (0.64 - 0.85) *p* <0.001
SYNTAX	2013	1800 (897 CABG vs. 903 PCI) Follow up 5 years	Compare CABG with PCI for treatment of patients with left main coronary disease or three-vessel disease.	Surgery remains the standard for patients with complex multivessel disease. In less complex disease PCI is a reasonable alternative treatment to CABG.	LVEF <30%: CABG 2.5% PCI 1.3%	SYNTAX Score ≥ 33: Death from cardiovascular causes CABG 14 (4.9%) vs. PCI 38 (13.6%) HR 2.99 (1.62 - 5.52) *p* 0.0002
SYNTAX	2019	1800 (897 CABG vs. 903 PCI) Follow up 10 years	Compare CABG with PCI for treatment of patients with left main coronary disease or three-vessel disease in 10 years follow up.	No significant differences in all cause death emerged between PCI with first generation paclitax eluting stents and CABG at 10 years. In patients with three vessel disease, CABG provided a significant survival benefit over PCI.	LVEF <30%: CABG 2.5% PCI 1.3%	SYNTAX Score ≥ 33: All cause death PCI 98/290 vs. CABG 82/315 HR 1.41 (1.05 - 1.89) *p* 0.30
HEART	2014	138 (69 conservative strategy vs. 69 invasive strategy)	Compare a conservative strategy with MT alone and without angiography vs. invasive strategy with an angiography with the intent to revascularize patients fit for CABG with heart failure and a wall motion index of <1.2, equivalent to an LVEF <35% and evidence of viable myocardium with impaired contractility.	Paucity of evidence that revascularization is appropriate for patients with heart failure and coronary artery disease other than for the relief of limiting angina even if there is a substantial amount of viable myocardium.	All population	Conservative strategy 25% vs. Invasive strategy 38%
Panza et al.	2014	1212 (Patients with 0-1 Prognostic Factors 576 vs. Patients with 2-3 636) Follow up 8 years	Examine the impact of anatomic variables associated with poor prognosis on the effect of CABG in ischemic cardiomyopathy.	Support the indication for surgical revascularization in patients with ischemic cardiomyopathy who present with more extensive CAD and worse myocardial dysfunction and remodeling.	All population	CABG vs. MT: 2-3 Prognostic factors: All cause mortality HR 0.71 (0.56 - 0.89) *p* 0.004 Cardiovascular death HR 0.72 (0.56 - 0.94) *p* 0.014 0-1 Prognostic factor: HR 1.08 (0.81 - 1.44) *p* 0.591, HR 0.89 (0.64 - 1.25) *p* 0.502
Wrobel et al. (Data from STICH)	2015	2136 patients (CABG + MT 1033 vs. CABG + MT + SVR 501 vs. MT 602) Follow up 30 days	1. Evaluate association of baseline patient characteristics and operative conduct on 30-day postoperative complications and mortality. 2. Evaluate incidence of postoperative complications and their association with 30-day mortality.	CABG can be performed with relatively low 30-day mortality in patients with severe LVD and ischemic heart failure. Despite this, serious complications are relatively common and occur before death in the majority of patients dying within 30 days of surgery. Greater renal dysfunctions, LVESVI, advanced age and preoperative atrial fibrillation or flutter are the strongest predictors of poor survival. Prolonged CPB time is the single operative characteristic predictive of poor early outcome.	All population	Early 30 day mortality: Without major complication <1% 1 major complication 14.3% 2 major complications 31% 3 major complications 33% ≥ 4 major complications 59%

*CABG* Coronary artery bypass grafts, *MT* Medical therapy, *LVEF* Left ventricular ejection fraction, *CAD* Coronary artery disease, *LVD* Left ventricular dysfunction, *SVR* Surgical ventricular reconstruction, *LVESVI* Left ventricular end-systolic volume index

## Revascularization strategies and indications

### Off-pump vs on-pump surgery

Patients undergoing CABG for LVD constitute a less studied subgroup, with studies beginning in 1990 [53, 54] leading to increased experience with this group and the development of various available revascularization techniques. Since then, the off-pump coronary artery bypass grafting (OPCABG) technique has shown promise in achieving complete revascularization [55]. Particularly in a high-risk patient subgroup [56], multiple studies have demonstrated positive outcomes of OPCABG in these patients [57–59]. This technique avoids obligatory global myocardial ischemia from aortic cross-clamping, cardioplegia arrest, and systemic inflammation, which are especially detrimental in high-risk patients [60]. This approach significantly reduces intraoperative organic damage due to the postcardiopulmonary bypass inflammatory response, particularly to the myocardium, kidneys, liver, and lungs, thereby improving outcomes in patients with preoperative organ dysfunction (see Table 3) [61].

**Table 3:** Evidence comparing ONCABG versus OPCABG in patients with Left Ventricular Dysfuntion

Study Title / First Author	Sample Size / Follow Up	Study Objective	Study Main Conclusion	Low Ejection Fraction	Mortality	Stroke	Perioperative AMI	Completeness of revascularization / Grafts per patient
Keeling et al. (2013) [55]	25667 (ONCABG 20509 vs. OPCABG 5158)	Evaluate outcomes of patients with LVD who underwent surgical revascularization with or without CPB.	Patients with EF ≤30% OPCABG yield superior short-term results compared with ONCABG strategies.	All population	In hospital death ONCABG 629 (3.1%) vs. OPCABG 158 (3.1%)	ONCABG 394 (1.9%) vs. OPCABG 68 (1.3%)	ONCABG 147 (0.7%) vs. OPCABG 22 (0.4%)	-
Darwazah et al. (2006) [57]	150 (66 OPCABG vs. 84 ONCABG)	Evaluate the difference in early morbidity and mortality among patients with LVD after myocardial revascularization using OPCABG or ONCABG.	The use of OPCABG resulted in better clinical outcome and mortality despite having a higher predicted risk score, but less number of grafts performed vs ONCABG. Patients with lowest EF ≤25% are better managed by an OPCABG, avoiding the effects of CPB.	All population	OPCABG 6.1% vs. ONCABG 10.7%, *p* 0.01	-	OPCABG 6.1% vs. ONCABG 2.4%	Completeness of revascularization ONCABG 85.7% vs. OPCABG 69.7%, *p* 0.01
Shennib et al. (2002) [58]	77 (OPCABG 31 vs. ONCABG 46)	Compare the outcome of OPCABG and ONCABG in patients with poor LVEF.	These patients may undergo surgical revascularization using OPCABG with relatively good results and low mortality levels. The lower number of grafts performed did not seem to affect clinical outcomes.	All population	OPCABG 3.2% vs. ONCABG 10.9% *p* 0.39	OPCABG 0 vs. ONCABG 4.3%	OPCABG 6.5% vs. ONCABG 4.3% *p* >0.99	Grafts per patient: OPCABG 2.8 + or - 0.8 vs. ONCABG 3.9 + or - 0.8 *p* <0.01
Emmert et al. (2011)	478 (OPCABG 256 vs. ONCABG 222)	Compare outcomes of OPCABG vs. ONCABG.	OPCABG in high risk patients with severely decreased EF is safe. It comes with similar mortality and MACCE and may benefit in regard to non-cardiac complications and is not at cost of less complete revascularization.	All population	OPCABG 1.4% vs. ONCABG 4.1%, OR 0.57 CI 95% (0.20 - 1.62) *p* 0.29	OPCABG 2.3% vs. ONCABG 2.7%, OR 0.86, IC 95% 0.28 - 2.72, *p* 0.80	OPCABG 1.4% vs. ONCABG 4.1%, OR 0.34 IC 95% 0.71 - 1.58, *p* 0.17	Completeness of revascularization: OPCABG 92.2% vs. ONCABG 92.8% Arterial grafts per patient 1.49 + or - 0.98 vs. 1.35 + or - 0.8 *p* 0.28
Woo et al. (2006) [60]	91 OPCABG (31 poor LVEF and 60 normal LVEF)	Evaluate potential benefits of OPCABG for patients with significant LVD compared with patients with normal LV function undergoing OPCABG.	Patients with significant LVD undergoing OPCABG demonstrated excellent perioperative outcomes that exceeded those previously reported and were equivalent to outcomes in our patients with normal LV function.	31 patients with LVEF ≤35%	-	-	-	Grafts per patient: Poor LVEF 2.8 + or - 0.1, Normal LVEF 2.9 + or - 0.1
Gaudino et al. (2004) [61]	306 (OPCABG 197 vs. ONCABG 109) Follow up 16 + or - 9 months	Verify if the theoretical benefits of OPCABG translate to effective clinical benefits in all complex CABG patients or at least in some subsets of them.	Adoption of OPCABG does not confer significant clinical advantages in all high risk CABG patients. Adaptation of the operation to the determinants of the operative risk of the single patient is likely to be the way to improve outcome.	LVEF ≤35% OPCABG 28/197 vs. ONCABG 34/109	ONCABG 7/109 vs. OPCABG 12/197, OR 0.83 CI 95% 0.01 - 9.72, *p* 0.9	ONCABG 1/109 vs. OPCABG 1/197, OR 0.76, CI 95% 0.08 - 9.98, *p* 0.91	ONCABG 4/109 vs. OPCABG 8/197, OR 0.93, CI 95% 0.04 - 10.12, *p* 0.99	Anastomoses per patient ONCABG 2.71 vs. OPCABG 1.79, OR 1.56, CI 1.12 - 4.89), *p* 0.03
Shroyer et al. (2009) [62]	2203 (OPCABG 1065 vs. ONCABG 1062) Follow up 1 year	Evaluate the primary outcomes of major morbidity and mortality at both 30 days and 1 year and the secondary outcomes of completeness of revascularization, 1-year graft patency, neuropsychological test scores and other outcomes.	Trial did not show any overall advantage to the use of OPCABG compared with ONCAB for coronary bypass. Instead there was a consistent trend toward better outcomes in patients with ONCABG, including better 1-year composite outcomes and 1-year patency rates.	LVEF ≤35% OPCABG 61/1065 vs. ONCABG 61/1062	Death within 30 days after surgery or before discharge: OPCABG 18 (1.6%) vs. ONCABG 13 (1.2%), RR 1.38, CI 95% 0.91 - 1.74) *p* 0.19 Death from cardiac causes within 1 year: OPCABG 29 (2.7%) vs. ONCABG 13 (1.3%) RR 2.05 CI 95% (1.09 - 3.86) *p* 0.03	OPCABG 14 (1.3%) vs. ONCABG 8 (0.7%), RR 1.75 CI 95% (0.74 - 4.14), *p* 0.28	Nonfatal AMI between 30 days and 1 year after surgery: OPCABG 21 (2%), ONCABG 23 (2.2%), RR 0.90 CI 95% (0.50 - 1.62), *p* 0.76	Patency of grafts: OPCABG vs. ONCABG: SVG 76.6% vs. 83.8% RR 0.91 CI 95% 0.88 - 0.95), *p* <0.001, LITA 95.3% vs. 96.2% RR 0.99 CI 95% (0.97 - 1.01), *p* 0.48, Overall 82.6% vs. 87.8% RR 0.94 CI 95% (0.92 - 0.97), *p* <0.001
Lamy et al. (2013) [63]	4752 (OPCABG 2375 vs. ONCABG 2377) Follow up 1 year	Evaluate the effects of OPCABG as compared with ONCABG.	At 1 year, no significant differences were found between the two groups in the rate of death, nonfatal stroke, nonfatal myocardial infarction or non fatal new renal failure requiring dialysis or in the rate of subsequent revascularization procedures.	LVEF ≤35% OPCABG 124 (5.4%) vs. ONCABG 131 (5.6%)	Death at 1 year: OPCABG 122 (5.1%) vs. ONCABG 119 (5%) HR 1.03 CI 95% (0.80 - 1.32), *p* 0.24 Cardiovascular related death: 99 (4.2%) vs. 96 (4%) HR 1.03 CI 95% (0.78 - 1.37) *p* 0.83	OPCABG 36 (1.5%) vs. ONCABG 40 (1.7%) HR 0.90 CI 95% (0.97 - 1.41), *p* 0.24	OPCABG 161 (6.8%) vs. ONCABG 178 (7.5%) HR 0.90 CI 95% (0.73 - 1.12), *p* 0.24	Repeat revascularization OPCABG 33 (1.4%) vs. ONCABG 20 (0.8%) HR 1.66 CI 95% 0.95 - 2.89) *p* 0.07
ROOBY-FS Quin et al. (2022) [64]	2203 (OPCABG 1065 vs. ONCABG 1062)	Investigate whether there may be long-term differences at 10-year clinical trial outcomes and post-CABG costs between OPCABG and ONCABG treatments.	ROOBY-FS documented slightly shorter revascularization-free survival among patients in OPCABG group. For most patients undergoing ONCABG who present as a viable candidate for either ONCABG or OPCABG no tangible long-term advantages were identified compared with traditional ONCABG.	LVEF ≤35% OPCABG 61/1065 (5.7%) vs. ONCABG 61/1062 (5.7%)	OPCABG 378 (34.2%) vs. ONCABG 342 (31.1%) RR 1.05 CI 95% (0.99 -1,11) *p* 0.12	-	-	Repeat Revascularization OPCABG 227 (20.6%) vs. ONCABG 210 (19.1%) RR 1.02 CI 95% (0.98 - 1.06) *p* 0.39
Puskas et al. (2009) [56]	14766 (7083 OPCABG vs. 7683 ONCABG) Follow up 30 days postoperative	Study which patients subgroups may benefit most from OPCABG rather than CABG on CPB.	OPCABG is associated with lower operative mortality than CABG on CPB in this single-institution retrospective study. This is driven by a disproportionate mortality benefit for higher risk patients, which increases with increasing STS PROM.	Not specified LVEF ≤50% OPCABG 38.1% vs. ONCABG 52%	Higher risk profile had a mortality benefit with OPCABG OR 0.62 and 0.45 for OPCABG for third and fourth risk quartiles Fourth quartile OR 0.45, CI 95% (0.33 - 0.63), *p*<0.0001	-	-	-

*ONCABG* On pump coronary artery bypass grafting, *OPCABG* Off pump coronary artery bypass grafting, *LEF* Low ejection fraction, *CPB* Cardiopulmonary bypass, *SVG* Saphenous Vein Graft, *LITA* Left internal thoracic artery

Subsequently, several studies have evaluated these two techniques in patients with ischemic cardiomyopathy and low LVEF , yielding diverse results. Shennib et al. [58] found no significant differences in outcomes or complete revascularization between 77 patients treated with LVD and those treated with either technique. The ROOBY trial [62] concluded that short-term outcomes did not differ between on-pump coronary artery bypass grafting (ONCABG) and OPCABG, but the long-term mortality risk was greater with OPCABG. However, the trial was criticized for its selection of surgeons who performed the procedures, and there was also an underrepresentation of patients with LVD, accounting for only 5.7% of the total population.

Keeling et al. [55] reported data from the STS database registry comparing 25,667 patients with LVD who underwent ONCABG (79.9%) versus OPCABG (20.1%). In the OPCABG subgroup, more comorbidities and a greater predicted preoperative mortality risk were observed, but a better incidence of distal anastomoses was noted with this technique. However, compared with those of ONCABG, lower rates of in-hospital mortality and postoperative neurological events and decreased prolonged ventilation were observed. These improved postoperative outcomes of OPCABG could be explained by the reduced need for intraoperative transfusions [65] and the absence of global ischemia as well as the shorter surgical time [55]. Despite the fewer distal anastomoses, recent data showed that, in older adults, surgery did not increase all-cause mortality given the preoperative risk profile [66].

In the CORONARY trial, 4752 patients randomized to OPCABG or ONCABG were evaluated, with 23% having LVD. A subgroup analysis comparing patients based on the EuroSCORE [63] revealed that, in low-risk patients (EuroSCORE 0-2), there was a trend toward worse 1-year mortality with OPCABG. Conversely, in high-risk patients (EuroSCORE ≥3), better outcomes were observed with OPCABG [67]. One possible interpretation is that in low-risk cases, events related to cardiopulmonary bypass (CPB) are rare, as are technical adverse events such as incomplete revascularization or a decrease in graft permeability. Conversely, in high-risk patients, adverse events related to CPB and aortic manipulation are more common, surpassing the risks associated with technical adverse events [68]. Based on this premise, they concluded that preferring ONCABG in low-risk patients and OPCABG in medium- to high-risk patients was reasonable [63].

When the ROOBY Follow-up Study was published [64], no advantage of OPCABG in terms of outcomes or costs was found after 10 years of follow-up. Slightly shorter revascularization-free survival times were observed, but no additional benefits were found in high-risk patients. The authors highlighted that both techniques are complementary and are part of the revascularization possibilities; however, there is no reason to prefer one technique over the other for candidates suitable for both techniques. Nevertheless, even in patients with extreme LVD (LVEF 10-20%), patients could undergo OPCABG with a reasonable mortality rate (11%) and achieve excellent long-term results, reaching an average LVEF of 35% at the 1-year follow-up, as described by Carr et al. [69]. Moreover, this technique is considered a viable option in special populations, such as patients on hemodialysis [70] or diabetic patients [71], with a high burden of aortic atherosclerotic disease and a high risk of stroke, where the use of the 'Aorta non-touch' technique [72, 73] under OPCABG minimizes postoperative cerebrovascular complications.

In the context of OPCABG, the use of a preoperative intra-aortic balloon pump (IABP) has also been described in high-risk patients, as this improves cardiac performance and facilitates access to target vessels while maintaining hemodynamic stability [74, 75]. The beneficial effects of IABP include reducing ventricular afterload, improving coronary diastolic perfusion, and enhancing subendocardial perfusion, all of which contribute to maintaining hemodynamic stability during surgery [76]. Additionally, there is reported redirection of blood flow to ischemic myocardial areas, improved graft flow post-bypass, decreased ventricular arrhythmias, and a decreased rate of low cardiac output postoperatively have been reported, thereby reducing end-organ dysfunction [77]. One of its downsides involves vascular complications related to IABP, which occur in up to 15% of high-risk patients, especially in women, diabetic patients, smaller patients, and those with peripheral vascular disease [78]. These complications could be mitigated by assessing the status of the thoracic and abdominal aortas through angiography or CT scans beforehand, maintaining coagulation levels for more than 150 seconds with heparin, and shortening the IABP duration by removing it immediately after the procedure, whenever possible [76].

It is important to consider the technical aspects of OPCABG surgery to facilitate procedural efficacy. Among the most discussed are intracoronary shunts and the CO2 blower. Intracoronary shunts are utilized to sustain blood flow in the distal circulation while constructing coronary anastomoses, thereby preventing inadvertent suturing of the posterior walls. However, proximal vessel occlusion has improved time and visualization. Additionally, the use of humidified CO2 blowers aids in enhancing arteriotomy visualization, playing a critical role in the precise construction of anastomoses [68].

### Hybrid revascularization

For patients with multivessel coronary disease, both CABG and PCI are considered definitive treatments [79–81]. However, CABG has proven to be particularly effective in patients with left anterior descending artery (LAD) disease, owing to the established benefits of the left internal thoracic artery (LITA) graft to the LAD [82]. Hybrid coronary revascularization (HCR) combines the use of LITA-to-LAD anastomosis with PCI intervention for other non-LAD diseased coronary arteries [83].

The HCR is based on the known long-term benefits of CABG with LITA grafts to the LAD in patients with severe and complex LAD disease, as well as the advantages of PCI over saphenous grafts for non-LAD lesions [84]. HCR is described in two distinct ways: one involving simultaneously performing both procedures in a hybrid operating room and the other through staged approaches (see Table 4). Better outcomes are attributed to simultaneous complete revascularization, as in staged approaches, the period of incomplete revascularization is prolonged, increasing the risk of adverse cardiovascular events [84].

**Table 4:** Comparison of simultaneous or staged HCR procedures [84]

Category	Simultaneous HCR	Staged HCR
Interventions	One intervention.	Multiple interventions.
Correction of complications	Identification of stent patency and immediate surgical correction of complications.	It does not allow immediate surgical correction.
Initiation of antiplatelet therapy	Delay in starting clopidogrel until internal thoracic artery (ITA) graft skeletonization.	Revascularization performed without dual antiplatelet therapy (RVM-first approach).
Intervals of ischemia	Lower risk of longer ischemia.	Extended intervals of ischemia.
Complete revascularization	Immediate complete revascularization if successful.	Concerns related to incomplete revascularization.

There is limited data on the durability of HCR compared to that of conventional CABG. In a multicenter observational study, Harskamp et al. [85] compared mortality and complications between HCR and CABG in patients from The Society of Thoracic Surgeons (STS) Adult Cardiac Surgery Database, including 198,622 patients (0.48% HCR and 98.5% CABG), finding no significant differences in mortality or the composite of in-hospital mortality and complications. Similarly, Harskamp et al. [86] conducted a meta-analysis of 6 studies (1 case-control study and 5 propensity score-adjusted studies) involving 1,190 patients (30.8% HCR and 69.2% CABG), again revealing no differences in mortality or in the composite of in-hospital mortality and complications, however, HCR showed higher rates of repeat revascularization at 1 year. Zhu et al. [87] also performed a meta-analysis of 10 cohort studies involving 6,176 participants and found no differences in major adverse cardiovascular or cerebrovascular events or mortality up to the 1-year follow-up.

Hannan et al. [83] conducted a multi-institutional study involving 37,589 patients with multivessel disease (0.80% HCR and 99.2% CABG). The authors reported that HCR is seldom used as an alternative to CABG in patients with multivessel disease. Furthermore, after propensity score matching, they found that at 6 years, HCRs were associated with increased mortality (19.1% vs 14.2%) and a greater rate of repeated revascularization in the LAD artery, as well as in areas where percutaneous intervention was performed (23.4% vs 11.8%).

In fact, HCR might be an acceptable approach in the short term (1 year), especially when performed simultaneously. In the short term, patients have a lower risk of major adverse cardiovascular events (MACEs), including mortality, stroke, and myocardial infarction. Likewise, when performed in a scheduled manner, there is a reduced risk of transfusions and repeated revascularization. However, when evaluating long-term outcomes (6 years), a higher mortality rate and an increased need for repeated revascularization compared to CABG become evident (see Table 5) [84].

**Table 5:** Evidence supporting hybrid revascularization in LVD

Study Title / First Author	Sample Size / Follow Up	Study Objective	Study Main Conclusion	Low Ejection Fraction	Mortality	Stroke	Perioperative AMI	Repeat revascularization / Graft type
Hannan et al. (2020) [83]	37859 (303 HCR vs. 37556 CABG) Median Follow up HCR 3.72 years CABG 4.21 years	Compare HCR and CABG surgery medium-term outcomes	HCR is rarely performed as an alternative to conventional CABG in patients with CAD involving the LAD artery and at least one major epicardial artery. HCR is associated with higher mortality after 1 year and higher rates of repeat revascularization.	LVEF <29% HCR 1.32% vs. CABG 1.32%	Survival at 6 years HCR vs. CABG 80.9% vs. 85.5%, HR 1.48 CI 95% (0.94 - 2.33) After propensity match AHR 1.44 CI 95% (0.90 - 2.31)	-	-	Freedom of repeat revascularization at 6 years CABG 88.2% vs. HCR 76.6%, HR 2.22 (1.44 - 3.42) After propensity match AHR 2.21 (1.44 - 3.41)
Harskamp et al. (2014)	199572 (HCR 950 vs. CABG 198622) Follow up not specified	Assess and compare clinical and operative characteristics between HCR and conventional CABG Describe and compare in-hospital outcomes between HCR vs. CABG.	Patients who undergo HCR are sicker but have less extensive coronary disease. In comparison both concurrent and staged HCR more frequently involved MICS and less use of CPB. Adjusted in-hospital outcomes were overall comparable.	Not specified	Isolated CABG vs. Staged HCR vs. Concurrent HCR: Operative mortality 1.5% (2984) vs. 1.4% (11) OR 0.74 (0.42 - 1.30) *p* 0.29; vs. 3.6% (5) OR 2.26 (0.99 - 5.17) *p* 0.053	Isolated CABG vs. Staged HCR vs. Concurrent HCR: 1.2% (2446) vs. 0.5% (4) OR 0.35 (0.15 - 1.07) *p* 0.069 vs. 1.4% (2) OR 1.16 (0.30 - 4.49) *p* 0.83	-	Reoperation: Isolated CABG vs. Staged HCR vs. Concurrent HCR: 2.2% (4354) vs. 2.4% (19) OR 1.06 (0.68 - 1.66) *p* 0.80 vs. 3.6% (5) OR 1.58 (0.66 - 3.78) *p* 0.30

## Graft options for patients with ICM

Surgical revascularization seems to be the optimal choice for these patients. However, there are no international guideline recommendations regarding which grafts provide the greatest benefit to these patients or increase the likelihood of better outcomes. Therefore, a search was conducted for available information (see Table 6).

**Table 6:** Evidence supporting graft options in patients with LVD

Study Title / First author	Sample Size / Follow up	Low Ejection Fraction	Mortality	Perioperative AMI	DSWI	Graft type	Patency	Off pump
ART Taggart et al. (2019) [26]	3102 (1548 BITA vs. 1554 SITA) Follow up 10 years	Not specified	Death at 10 years: BITA 20.3% vs. SITA 21.2%, HR 0.96 CI 95% (0.82 - 1.12) *p* 0.62	BITA 4.6% vs. SITA 5.0%, HR 0.92 CI 95% (0.66 - 1.26) *p* 0.62	Sternal wound complication: BITA 2% vs. SITA 0.6% HR 1.81 CI 95% (1.16 - 2.86)	Additional RA grafts: BITA 19.4% vs. SITA 21.8% Mortality single vs. multiple arterial grafts: BITA vs. SITA HR 0.81 CI 95% (0.68 - 0.95)	-	BITA 41.9% vs. SITA 40%
Lytle et al. (2004) [88]	2304 (SITA 1152 vs. BITA 1152) Follow up 20 years	Severe LVD: SITA 23% vs. BITA 24%	Survival at 7, 10, 15 and 20 years BITA vs. SITA: 89% vs. 87%, 81% vs. 78%, 67 vs. 58% and 50% vs. 37% respectively *p* <0.0001	-	-	-	-	-
Galbut et al. (2012) [89]	4537 (SITA 2340 vs. BITA 2197) Follow up 30 years	LVEF <30%: SITA 11.6% vs. BITA 6.9%	Operative mortality in LVEF <30%: SITA 10.3% vs. BITA 6.9%, *p* 0.418 Survival at 7 and 14 years in LVEF <30% SITA vs. BITA: 48.3% SD 4.2% vs. 51.7% SD 5.4% and 20.7% SD 3.6% vs. 26.7% SD 4.9% respectively, *p* 0.289	LVEF <30%: SITA 5.7% vs. BITA 1.1%, *p* 0.097	LVEF <30%: SITA 2.3% vs. BITA 2.3% *p* 1.000	-	-	-
Ariel et al. (2020)	394 (BITA 206 vs. SITA 188) Follow up 14 years	All population (52.2% in BITA vs. 47.8 in SITA)	30 day mortality: SITA 8.5% vs. BITA 6.8%, *p* 0.55 Survival at 1, 3, 5, 10 years SITA vs. BITA: 84.1% vs. 86.5%, 76.7% vs. 75.9%, 66.1% vs. 69.4% and 45.8% vs. 46.5% respectively, *p* 0.36	SITA vs. BITA: Unmatched analysis 8.5% vs. 6.8% *p* 0.55 Matched analysis 3.9% vs. 2.3%, *p* 0.727	SITA vs. BITA: Unmatched analysis 2.7% vs. 1%, *p* 0.266 Matched analysis 1.6% vs. 0.8%, *p* >0.999	SITA vs. BITA: SVG 77.7% vs. 26.7%, *p* <0.001 RA 19.1% vs. 2.4%, *p* <0.001 GEA 6.9% vs. 16%, *p* 0.005	-	SITA vs. BITA: 26.6% vs. 15.5%
Mohammadi et al. (2014) [90]	1795 (BITA 129 vs. SITA 1666) Follow up median 7.5 years	LVEF <30%: BITA 30.2% vs. SITA 34.5%	BITA vs. SITA: Operative mortality 1.8% vs. 0.9%, *p* 0.6 Survival at 5, 10 and 15 years: 93.7% vs. 82.8%, 77.5% vs. 68.1% and 59% vs. 65.2%, respectively, *p* 0.6 BITA vs. SITA for mortality: HR 0.7 CI 95% (0.4 - 1.3), *p* 0.3	BITA vs. SITA: Unmatched analysis 2.4% vs. 4.5%, *p* 0.53 Matched analysis 2.5% vs. 2.5%, *p *1	BITA vs. SITA: 3.1% vs. 2.6%, *p* 0.72 Mortality with DSWI: HR 7.4, CI 95% (2.2 - 24.1), *p* 0.001	-	-	-
Kelly et al. (2012) [91]	8264 (No ITA 631 vs. SITA 6554 vs. BITA 1079) Median Follow up 4.7 years	LVEF <40% No ITA vs. SITA vs. BITA: 24% vs. 12% vs. 7%	No ITA vs. SITA vs. BITA: Hospital mortaity 7.3% vs. 2.1% vs. 1.7%, *p* <0.0001 Long-term mortality 48.3% vs. 21.9% vs. 12.3%, *p* <0.0001	No ITA vs. SITA vs. BITA: 1.1% vs. 1.1% vs. 0.4%, *p* 0.067	No ITA vs .SITA vs. BITA: 0% vs. 0.7% vs. 1.2%, *p* 0.014	No ITA vs. SITA vs. BITA: SVG 100% vs. 89% vs. 36%, LITA 0% vs. 99% vs. 100%, RITA 0% vs. 1% vs. 100%, GEA 0% vs. 0.2% vs. 100%	-	-
Kurlansky et al. (2010) [92]	4584 (SITA 2369 vs. BITA 2215) Follow up 30 years	LVEF <30% SITA 6.2% vs. BITA 3.9%	SITA vs. BITA Hospital mortality 4.6% vs. 2.6%, *p* 0.001 Survival at 15 and 25 years: 37.5% vs. 53.5% and 15.7% vs. 28.6%	SITA vs. BITA 5.7% vs. 4.8% *p* 0.016	SITA vs. BITA: 1.1% vs 4.8%, *p* 0.289 Adjusted with diabetic population: 1.0% vs. 2.8% *p* <0.003	-	-	-
Munakata et al. (2020) [70]	63 (BITA 30 vs. SITA 33) Follow up 6 years	BITA 30% vs. SITA 33.3%	BITA vs. SITA: Hospital death 0 vs. 3% *p* 0.336 Cardiac deaths during follow up 8 (67%) vs. 12 (52%)	-	BITA vs. SITA: 3.3% vs. 3%, *p* 0.945	RCA revascularization: BITA 12 (40%) vs. SITA 21 (63.6%) GEA to RCA: 12 (40%) vs. 5 (15.2%) SVG to RCA 5 (16.7%) vs. 16 (48.5%)	Early graft patency: BITA vs. SITA 1/103 (0.97%) grafts ocluded (SVG-RCA) vs. 3/92 (3.3% occluded (RITA-LAD 1, LITA-LCX 2) 3 years patency: ITA patency stimate 86.2%	BITA vs. SITA: CPB use 33.3% vs. 45.5% p 0.214 Aorta non-touch technique BITA 45.7%
Calafiore et al. (2004) [93]	1602 (SITA to LAD + SVG 576 vs. BITA to LAD 1026) Follow up 10 years	LVEF ≤35% 4.4% vs. 5.3%	BITA vs. SITA: 2.1% vs. 2.8% *p* 0.444 Cardiac deaths 1.2% vs. 2.3%, *p* 0.176 Freedom from cardiac death after 10 years: BITA 96.5% vs. SITA 91.3%, *p* 0.0288	BITA vs. SITA: AMI 0.5% vs. 1.6% 0.082 Non-fatal AMI 0 vs. 0.4% *p* 0.396 AMI in grafted areas after 10 years: 98.4% vs. 94.7% *p* 0.0057	-	-	-	BITA vs. SITA: 32.5% vs. 24.2%
Hirotani et al. (2003) [94]	303 (BITA 179 vs. SITA 124) Follow up 10 years	LVEF ≤30% BITA 84% vs. SITA 82%	BITA vs. SITA: Operative mortality 1.6% vs. 1.7% Insuline treated: 1.2% vs. 1.6% Not insuline treated 2.2% vs. 1.6%	BITA vs. SITA: 1.1% vs. 1.6%, *p* 0.71	Wound infections total: BITA 8.4% vs. 6.5% Insuline treated: BITA 10% vs. 13% Not insuline treated 6.5% vs. 0%	BITA vs. SITA: Mean number of additional SVG 1.26 vs. 1.65, NS Mean number of additional GEA grafts 0.045% vs. 0.081%, NS	Early grafts patency ITA 98.5% (Right ITA 97%, Left ITA 99.6%) SVG 95.4%	-
Endo et al. (2001) [95]	1131 (433 BITA vs. 688 SITA) Follow up 7 years	LVEF ≤40% BITA 18.7% vs. SITA 20.1% LVEF ≤40% associated with mortality HR 2.47 (1.77 - 3.44) *p* 0.0001	BITA vs. SITA: 0.9% vs .0.9%, *p* 0.999 Long term cardiac mortality 3.2% vs. 5.1%	BITA vs. SITA 2.5% vs. 2.8%, *p* 0.776	BITA vs. SITA 0.6% vs. 0.5% *p* 0.999	BITA Group: 1283 anastomoses, 13.5% GEA, 1.2% RA, 0.1% IEA SITA Group: 1828 anastomoses, 13.2% GEA, 1.1% RA, 0.1% IEA	Early graft patency BITA group 97.3% vs. SITA group 94.3% Patency ITA 98%, GEA 94.6%, SVG 91.7%	-
Toumpoulis et al. (2006) [96]	980 (490 BITA vs. 490 SITA) Mean Follow up 4.7 years	LVEF ≤30% BITA vs. SITA 20.6% vs. 19.4%	BITA vs. SITA: 30 day mortality 3.9% vs. 3.7%, *p* 0.999 In hospital mortality 3.9% vs. 2.9%, *p* 0.479	BITA vs. SITA 0.8% vs. 0.6%, *p* 0.726	BITA vs. SITA: 3.3% vs. 1.2%, *p* 0.050	-	-	BITA vs. SITA: 5.5% vs. 7.1%
Joo et al. (2012) [97]	1749 (BITA 392 vs. SITA 1357) Follow up 10 years	BITA vs. SITA 0.115% (45) vs. 0.105% (142)	BITA vs. SITA operative mortality 0.5% vs. 0.8%, *p* >0.99	BITA vs. SITA 0.5% vs. 0.5% *p* >0.99	-	BITA vs. SITA Use of vein graft 0.27 vs 0.59, *p* <0.001 Total arterial grafting 91.5% vs. 79.5%, *p* <0.001	-	All population off pump
Matsa et al. (2001) [71]	765 (Diabetic 231 vs. Non-diabetic 534) Follow up 3 years	LVEF <36% Diabetic vs. Non-diabetic 19.9% vs. 18%	Diabetic vs. Non-Diabetic Operative mortality 3% vs. 2.6%, *p* 0.80 Three-year survival 93% vs. 94.7%, *p* 0.08	Diabetic vs. Non-diabetic 0 vs. 1.7%, *p* 0.06	Diabetic vs. Non-diabetic 2.6% vs. 1.7%, *p* 0.40	Diabetic vs. Non-diabetic: GEA 19.4% vs. 24%, SVG 11.2% vs. 12%	-	-
Locker et al. (2012) [98]	8622 (LITA + SVG 7435 vs. Multiarterial 1187) Follow up 15 years	Not specified	LITA + SVG vs. Multiarterial Operative mortality 0.8% vs. 2.1%, *p* 0.005	-	-	LITA + SVG 7435, Multiarterial included 5 subgroups BITA+SVG 589, BITA 271, BITA+RA 147, LITA+RA 169 BITA+RA+SVG 8	-	LITA+SVG vs. Multiarterial 4.4% vs. 3.3%
Kinoshita et al. (2012) [99]	434 (BITA 217 vs. SITA 217) Follow up 5 years	LVEF ≤40% BITA 26.3% vs. SITA 23.6%	BITA vs. SITA In hospital mortality 2.8% vs. 3.2%, *p* 0.79 30-day mortality 2.3% vs. 1.8%, *p* 0.74	BITA vs. SITA 1.4% vs. 0.9%, *p* 0.43	BITA vs. SITA 0.9% vs. 1.4%, *p* 0.43	-	-	All population off pump
Tsuneyoshi et al. (2015) [73]	384 (232 BITA vs. 152 LITA + RA) Follow up 14 years	LVEF ≤40% 3% vs. 2%	BITA vs. SITA Hospital mortality before matched analysis 0.9% vs. .07%, *p* 0.7 After matched analysis 0.8% vs. 0, *p* 0.38	BITA vs. LITA + RA 1.3% vs. 1.3%, *p* 0.87 After propensity matching 1.7% vs. 1.7%, *p* 1.0	BITA vs. LITA + RA 2.1% vs. 0, *p* <0.01 After propensity matching 2.5% vs. 0, p<0.01	BITA 232 vs. 152 LITA + RA	Long term patency (5 years) RITA 78% vs. RA 84%, *p* 0.55	All population off pump
Gaudino et al. (2018) [100]	1036 (536 RA vs. 502 SVG) Follow up 5 years	LVEF <35%: RA 25 (4.7%) vs. SVG 32 (6.4%)	RA vs. SVG: No of events 40 (7.5%) Events per 1000 Patient-year 15 vs. 42 (8.4%) Events per 1000 patients-year 17, HR 0.90 (0.59-1.41) *p* 0.68	RA vs. SVG: No of events 16 (3%) Events per 1000 patients-year 6 vs. No of events 21 (4.2%) Events per 1000 patients-year 9, HR 0.72 (0.53 - 0.99) *p* 0.04	-	534 radial artery grafts 502 saphenous vein grafts	RA vs. SVG: Graft occlusion 28/345 (8.1%) vs. 61/307 (19,9%), HR 0.44 (0.28 - 0.70) *p* <0.001 Repeat revascularization: 24 (4.3%) vs. 43 (8.6%), HR 0.50 (0.40 - 0.64) *p* <0.001	
Tranbaugh et al. (2014) [101]	1056 (528 RA vs. 528 RITA to LCX) Follow up 15 years	LVEF <35% population not specified but in SD included	RA vs. RITA: Hospital and 30 day mortality 0.6 vs. 1.7%, *p* 0.082 All-cause mortality: 13.7% vs. 30.2%, *p* <0.0001 Early and late all cause mortality: 17% vs. 22.5%, *p* 0.025	RA vs. RITA: 1.1% vs. 0.6%, *p* 0.315	RA vs. RITA: 1.1% vs. 2.7%, *p* 0.071	528 RA graft vs. 528 RITA grafts Arterial Grafts per patient: RA 2.4 + or - 0.6 vs. 3.1 + or - 0.7	Patency after mean time of 5 years: RA 83.9% vs. RITA 87.4%, *p* 0.155 LITA 92.7%, SVG 56.7%	All on pump
Caputo et al. (2003) [102]	661 (RITA 336 vs. RA 325) Follow up 1 year	LVEF Fair or poor: RITA 46 (13.8%) vs. 85 (26.2%)	RITA vs. RA: Deaths in the hospital 3 (0.9%) vs. 1 (0.3%) OR 0.34 CI 95% (0.03 - 3.30) *p* 0.35 Deaths during follow up 5 (1.5%) vs. 1 (0.3%)	RITA vs. RA: 7 (2.1%) vs. 1 (0.3%) OR 0.15 (0.02 - 1.03) *p* 0.05	-	RITA 335 vs. Radial artery 325	RITA vs. RA: Repeat revascularization due to occlusion 3 (0.9%) vs. 3 (0.9%)	RITA vs. RA: On pump 258 (76.8%) vs. 197 (60.6%)
Navia et al. (2014) [103]	1700 (1447 BITA vs. 253 LITA + RA) Follow up 8 years	BITA vs. LITA + RA: Severe LVEF 19.7% vs. 35%	BITA vs. LITA + RA: 37 deaths due to cardiac causes (24 vs. 13) *p* 0.08 Postoperative survival at 1, 4 and 7 years: 99%, 94% and 90% vs. 97%, 91% and 83% *p* 0.02 HR 0.59 (0.38 - 0.92) *p* 0.021	-	-	-	-	All population off pump
RAPCO Trial Long-Term Buxton et al. (2020) [104]	394 (198 RA vs. 196 free RITA) Follow up 10 years	LVEF not specified	RA vs. RITA Deaths during follow up 18 vs. 32 Ten-year patient survival estimate 90.9% vs. 83.7%, HR 0.53 CI 95% (0.30 - 0.95) *p* 0.03	RA vs. RITA during follow up 11 vs. 13 AMI events HR for MACE 0.65 CI 95% (0.44 - 0.96)	RA vs. RITA: DSWI events 0 vs. 2, *p* 0.24	Radial artery group 198 vs. free RITA group 196	RA vs. RITA: 10 year patency 89 vs. 80%, HR 0.45 (0.23 - 0.88)	-
RAPS Deb et al. (2012) [105]	510 patients with multivessel disease with grafts of RA and SVG Follow up 5 years	Not specified LVEF	<30 days: 3 (0.6%), 31 days to 1 year 5 (0.9%), 1 year to last follow up 53 (10%), total events 61 (11.5%) Death from cardiac causes <30 days 1 (0.2%), 31 days to 1 year 4 (0.8%), 1 year to last follow up 21 (4%)	<30 days 53 (10%), 31 days to 1 year (0.2%), 1 year to last follow up 9 (1.7%), total events 63 (11.9%)	-	234 Radial artery grafts vs. 234 Saphenous vein graft	RA vs. SVG: Functional graft occlusion 12% vs. 19.7%, *p* 0.003 Complete graft occlusion 8.9% vs. 18.6%, *p* 0.002	-
Jung et al. (2009) [106]	1735 (Group 1 451 RA direct to aortic anastomosis vs. Group 2 442 RA composite grafting with ITA) Follow up 6 years	Group 1 vs. Group 2 LVEF≤35% 4.8% vs. 6.7%	Group 1 vs. Group 2 Early mortality 0.86% vs. 0.82%, *p* 0.932	-	-	451 radial artery graft direct aortic anastomosis vs. 442 radial artery composite grafting with ITA	RA graft patency at 1, 2 and 5 years: Group 1 93.8%, 90.5% and 74.3% vs. Group 2 90.5%, 85.3% and 65.2% respectively	Group 1 26% vs. Group 2 42.6%

*BITA* Bilateral internal thoracic artery, *SITA* Single internal thoracic artery, *RA* Radial artery, *LVD* Left ventricular dysfunction, *SVG* Saphenous vein graft, *GEA* Gastroepiploic artery, *RITA* Right internal thoracic artery, *RCA* Right coronary artery, *LAD* Left anterior descending artery, *LCX* Left circumflex artery, *IEA* Inferior epigastric artery

Performing CABG using a LITA graft is an effective approach for treating patients with advanced and symptomatic CAD [26] and has shown superiority to PCI in severe CAD and diabetic patients [27]. This superiority is attributed to better long-term patency and survival than those of saphenous vein grafts (SVGs) [28]. The latter are mainly responsible for graft failure and exhibit intimal fibrosis and accelerated atherosclerosis, which can reduce graft patency by up to 50% at 10 years, compromising long-term outcomes [26, 29, 30]. Hence, exploration of the use of CABG with multiple arterial grafts has continued, as such use significantly impacts mortality [26]. These arterial grafts are living tissue that displays adaptive mechanisms increasing blood flow and shows resistance to atherosclerosis through nitric oxide release, as well as protection against proximal CAD progression [107, 108].

LVD is strongly associated with perioperative mortality [109]. The priority in these patients is to mitigate surgical risk [110]. The use of multiple arterial grafts may increase the complexity and duration of surgery, which may not be well-tolerated by patients with LVD [111] and is associated with increased mortality [52]. However, retrospective analyses have shown comparable operative safety between bilateral internal thoracic artery (BITA) use and single internal thoracic artery (SITA) use. Observational studies present conflicting information regarding long-term survival with BITA in this patient population [88–90, 112]. Although BITA is not routinely employed in LVD patients, it could be considered in selected scenarios guided by anticipated patient survival and the judgment and experience of the surgeon.

The use of the BITA strategy, utilizing the right internal thoracic artery (RITA) as a second arterial conduit, has been shown to be an independent survival factor compared to the use of the SVG [91]. In nearly all patients where BITA is technically feasible, a significant long-term survival benefit has been observed, leading to freedom from recurrent myocardial infarction and angina for up to 15 years [72, 92, 113]. An observational study from the Cleveland Clinic by Lytle et al. [88], as well as a meta-analysis of observational studies by Yi et al. [114], demonstrated that survival curves between SITA and BITA start diverging in favor of BITA revascularization at the 10th year of follow-up. Consequently, the hemodialysis patient group did not show apparent survival benefits with BITA grafting due to their relatively short life expectancy [70]. Its maximum benefit is achieved when using the RITA as a graft to the lateral wall rather than the right coronary artery (RCA) [93]. The only randomized study, the ART trial, comparing LITA + SVG vs. BITA, showed no significant differences between the two groups in terms of the rate of death from any cause (adjusted HR, 0.81 [95% CI, 0.68–0.95]) or composite of death, myocardial infarction, or stroke (adjusted HR, 0.80 [95% CI, 0.69–0.93]). However, potential confounding factors, such as the use of the radial artery (RA) as the second conduit in the SITA group, high adherence to guideline-directed medical therapy, and short follow-up time, were considered [26].

However, its use has not been universally accepted due to the association of BITA usage with an increased risk of sternal complications, including deep sternal wound infections (DSWI) and poor sternal healing due to decreased sternal perfusion [91, 94–97]. This risk is even greater in older patients, women, diabetic individuals and morbidly obese patients with a glycosylated hemoglobin (HbA1c) level >7.5%, with up to a 10-fold increase in the risk of DSWI [71, 115]. This risk has been mitigated by using the skeletonized BITA grafting technique to preserve sternal perfusion via collateral vessels [116], as well as by strictly controlling perioperative glucose levels through insulin infusions [96, 117, 118]. Hyperglycemia in the first two postoperative days is considered an independent predictor of DSWI development [118]. Multiple studies have shown that maintaining glucose levels <180 mg/dL with insulin infusions during surgery and in the early postoperative days, along with skeletonization, reduces the incidence of DSWI [118–120]. However, perfusion disturbances persist, as confirmed by SPECT perfusion tests, indicating a potential risk of sternal infection, especially in elderly and diabetic patients [116].

CABG with BITA is typically limited to patients <75 years old with suitable coronary targets [72]. Age is considered a limiting factor due to increased osteoporosis incidence, a greater proportion of diabetic patients, and a greater risk of stroke [121]. However, multiple studies have shown no differences in long-term survival up to the age of 79 years [30, 92, 96, 98, 99]. For high-risk patients, particularly those who are diabetic and elderly, a non-touch off-pump technique is considered, where avoiding aortic manipulation reduces the risk of postoperative stroke [72, 73]. This technique also decreases operative mortality and morbidity, especially in elderly patients or those with poor cardiac function [99].

Similarly, the use of the RA as a second or even third arterial conduit in an all-arterial revascularization strategy has been proposed. It is recommended to harvest from the side with better ulnar compensation and arterial quality, rather than from the nondominant side. Harvesting is usually well tolerated, with possible self-limiting and transient occurrence of paresthesia and pain [122]. Contraindications for its use include upper extremity vascular diseases; prior forearm trauma, especially if it requires surgical repair; and previous forearm or wrist surgeries [111]. Furthermore, in patients with chronic kidney disease, the potential benefit of CABG with an RA graft should be weighed against the possible need for an upper limb arteriovenous fistula for dialysis, although limited evidence exists to guide this decision [111].

Compared to SVG, the RA was associated with a significantly lower incidence of adverse cardiac events (HR 0.67, 95% CI 0.49 - 0.90) and a reduced incidence of occlusion (HR 0.44, 95% CI 0.28 - 0.70), with improved patency at 5 years [100]. RA is considered an alternative for mitigating or reducing the risk of DSWI associated with BITA [98]. Several authors have reported that RA is superior to RITA [101, 102], while others have indicated the opposite [103]. However, when comparing RA to RITA, there are no differences in graft patency, survival rate, or event-free survival rate, but differences exist in DSWI incidence and operative time, which tend to be longer with BITA [73]. Nevertheless, there was an underrepresentation of patients with LVD, with only 3% of patients having a low LVEF. Compared to SVG, RA exhibits superior patency and better clinical outcomes at 5 years [52]. There are RCTs such as the RAPCO Trial [104], which compares RA, BITA, and SVG patency at 10 years, and the RAPS [105], which compares RA and SVG patency at 5 years. However, these trials lack representation of patients with LVD, making their findings challenging to apply in this patient population. Jung et al. [106] demonstrated better patency rates when RA was used as a graft when directly anastomosed to the ascending aorta than when it was anastomosed to the side of the LITA, with patency rates of 85.3% vs. 65.2%, respectively, at 5 years. According to the most recent myocardial revascularization guidelines, the use of the RA for severe stenosis targets is proposed as a Class I recommendation with Level B evidence, while the BITA is a Class IIa recommendation with Level B evidence [10, 123]. Additionally, lesser-used grafts, such as those from the gastroepiploic artery (GEA) or the inferior epigastric artery (IEA), have been employed as third conduits, albeit less frequently and in nonmain branches [91, 94, 95, 97, 99]. Concerning all-arterial revascularization, significantly longer survival has been observed in patients receiving 3 arterial conduits than in those receiving 2 or 1 arterial conduit (HR 0.8, 95% CI 0.75 - 0.87), attributed to their greater long-term patency and lower association with major cardiovascular events [123].

In conclusion, according to multiple studies, there is an underrepresentation of the population with LVD, constituting a very small portion of the total population and being an exclusion criterion in some studies. However, these patients also benefit from using CABG with a second arterial conduit, either BITA or RA revascularization [92, 98]. This subset includes those who benefit the most from arterial revascularization [88], This incremental benefit becomes apparent early after surgery and persists during follow-up [99]. Performing OPCABG with in situ left-sided skeletonized BITA grafting is safe and feasible, with a decreased association with operative mortality and morbidity in patients with low cardiac function [56, 68, 99].

As previously indicated ICM accounts for more than 60% of the congestive heart failure cases, associated with high morbidity and mortality. However, this condition is not necessarily irreversible [2–4]. Revascularization of ischemic myocardial segments can lead to an improvement in overall LV function and consequently, better clinical outcomes [3–5]. Numerous studies have shown that revascularization can result in an improvement in LVEF in up to 60% of patients, with a wide range of improvement observed [15–29]. The objective of this review was to explore the variations in surgical techniques and graft options for myocardial revascularization of LVD patients that lead to the aforementioned LVEF improvement. This prompts the question: What is the clinical significance of an improvement in LVEF for patient-important outcomes? These outcomes are defined as “a characteristic or variable that reflects how a patient feels, functions or survives” [124–126]. As previously stated in ICM patients the treatment goals are prolonging survival, enhancing quality of life and reducing the risk of cardiac and non-cardiac complications.

The latest consensus statement of a universal definition for HF written by the Heart Failure Society of America (HFSA), Heart Failure Association of the European Society of Cardiology (HFA/ESC) and the Japanese Heart Failure Society (JHFS) created a novel entity of patients with heart failure with improved ejection fraction (HFimpEF). These were originally patients with LVD with a second measurement of LVEF >40% and a ≥10% increase from baseline LVEF of ≤ 40% [127]. Some studies show that HFimpEF patients had a better prognosis and a significant improvement in health-related quality of life [128, 129], however other concluded that the difference in the risk of death between patients with HFimpEF and heart failure with reduced ejection fraction (HFrEF) groups was not statistically significant [130] In a meta-analysis from He at al. [131] nine studies of 9491 heart failure patients were included and during a follow-up of 3.8 years, the pooled prevalence of who develop HFimpEF after treatment was 22.64%. HFimpEF reduces the risk of all cause mortality by 56%, cardiac hospitalization by 60% and composite events by 44% compared to patients with HFrEF. However this study had several limitations with an absence of a unified definition of HFimpEF, the lack of enough data to perform a subgroup analysis and the absence of randomized controlled trials.

In more recent studies, Wohlfart et al. [129] showed that recovery of systolic function was associated with HF-associated quality of life (QoL) improvements and for each 10% increase in LVEF, the Kansas City Cardiomyopathy Questionnaire score improved by a mean (SD) of 4.8 土 1.6 (*p* = 0.003). Likewise, DeVore et al. [132] reported QoL improvements related to ≥10% increase in LVEF in patients with initial HFrEF with a mean change of 7.6 points (range 6.0 - 9.2) in the Kansas City Cardiomyopathy Questionnaire compared with 3.5 points (range 2.3 - 4.8) in the non-HFimpEF (*p* < 0.001). In a study from Zamora et al. [133] the HFimpEF group had shorter HF duration and a larger proportion of patients classified as NYHA I-II. Also the perceived QoL improvement in patients with HFimpEF was mainly related to the number of HF-related hospitalizations in the previous year and NYHA functional class. Consideration must be given to the fact that QoL is inherently subjective and can be influenced by numerous factors, such as age, sex, previous hospitalizations, diabetes and treatments. The physical dimension is very important, patients with more comorbidities or higher NYHA functional classes reported worse QoL scores [133].

Several limitations need to be acknowledged in our study. As previously mentioned the majority of RCT exclude patients with ICM. The scarcity of RCTs in the existing literature that fulfill the inclusion criteria made us recur to observational studies with an underrepresentation of ICM patients, susceptible to bias. Also, the lack of a universal definition of LVD patients among the studies, the complexity of the topic and the heterogeneity of outcomes reported in the existing literature made a comprehensive assessment challenging. Therefore the best approach to overview and synthesize the available evidence was to perform a scoping review. Further RCT studies are necessary to assess the potential benefits of the available surgical approaches and graft options described in the literature. Also further randomized studies are required to explore the clinical significance of an improvement in LVEF and its impact on patient-important outcomes. To our knowledge there is an ongoing RCT, the MASS VI VF, which includes patients with multivessel CAD, angina pectoris and LVD (≤35%) and ≥10% ischemia detected by myocardial scintigraphy. The objective of this study is to randomize 300 patients to myocardial revascularization surgery and 300 patients to medical treatment only in order to evaluate if myocardial revascularization contributes to a better prognosis compared with those treated with OMT [134].

Patients with LVD and LVEF ≤35% have shown improvement after surgical revascularization, as demonstrated in this article. This LVEF improvement has also been strongly correlated with enhancements in quality of life and reductions in mortality rates. Future studies should prioritize refining the methodologies of current published trials by addressing both clinical and imaging indicators of myocardial viability, as well as implementing long-term follow-ups of revascularized patients. It is also essential to determine the method of follow-up, whether it should involve clinical assessments or less invasive imaging modalities. The advancements in coronary CT and MRI hold promise for the future, potentially aiding in the evaluation of graft patency and providing accurate quantification of LVEF, while being less invasive. Regarding patients presenting with acute coronary syndrome and LVD with an LVEF ≤ 35%, this remains another topic for review. It is still unclear whether these patients should be analyzed within this group.

## Indications Table (Table 7)

**Table 7:** Indications for different surgical revascularization strategies and graft options

**Indications**			
**Type of Revascularization**	**Hybrid Revascularization **[7, 10, 83, 85, 87]	**CABG Revascularization **[7, 10]	**-**
	LAD lesion ≥70% and anatomy eligible for CABG. Non-LAD lesions ungraftable and amenable for PCI. Recommended in centers familiarized with minimal cardiac surgery approaches. Use in patients where the surgical risk needs to be minimized. Role in Patients with LVD is unclear. Heart Team discussion and individual prospective planning is necessary.	Patients with SIHD. Multivessel CAD with ≥ 70% stenosis. Left main CAD. With LITA graft to LAD artery and suitable saphenous veins to perform an SVG or absence of PAD to perform arterial grafts.	
**CPB Usage**	**ONCABG **[7, 10, 67]	**OPCABG **[55, 56, 67, 68, 77]	**-**
	Patients with SIHD. Multivessel CAD with ≥ 70% stenosis. Left main CAD. With LITA graft to LAD artery. Patients with low risk score (EuroSCORE 0-2).	Older patients. Female gender. More comorbidities (preoperative arrhythmia, COPD, Peripheral vascular disease, cerebrovascular disease, diabetes mellitus, preoperative renal dysfunction). Higher predicted risk of mortality score. Patients with moderate to high-risk score (EuroSCORE ≥ 3). To minimize risk of stroke, renal failure, blood transfusions, respiratory failure and development of atrial fibrillation. To minimize midterm cognitive dysfunction. Use of preoperative IABP in patients with severe LVD allows complete revascularization.	
**Type of Graft**	**SITA + SVG **[7, 10]	**BITA **[70, 72, 96, 111, 118–120]	**Radial Artery **[10, 70, 98, 111, 123]
	Peripheral artery disease. Patients with hemodialysis with the need to preserve arterial conduits for hemodialysis.	Patients <75 years old. Anticipated long term survival. Nondiabetic patients. Diabetic patients with perioperative glucose levels <180 mg/dL and HbA1C <7.5%. • Harvesting with the use of skeletonized technique. No anticipation to perform a redo sternotomy.	Diabetic patients. High risk patients for DSWI. Patients without upper extremity artery disease. Patients where it is anticipated the need of a redo sternotomy.

*SIHD* Stable ischemic Heart Disease, *CAD* Coronary artery disease, *LITA* Left internal thoracic artery, *LAD* Left anterior descending artery, *SVG* Saphenous vein graft, *PAD* Peripheral artery disease, *LEF* Low ejection fraction, *SITA* Single internal thoracic artery, *HbA1C* Glycosylated hemoglobin, *DSWI* Deep sternal wound infection

## Conclusions

In conclusion, the literature highlights the scarcity of ischemic left ventricular dysfunction patients in randomized studies, with observational studies predominating. However, existing data support the benefits of coronary revascularization in LVD, evidenced by improved LVEF and QoL.

ONCABG is recommended for multivessel disease in patients with LVD and LVEF < 35%, with LITA-to-LAD graft providing significant benefits. Angina and/or demonstrated myocardial ischemia are necessary for better outcomes.

OPCAB is proposed for older, higher-risk patients, with the aorta-non-touch technique reducing stroke risk. Preoperative IABP use may improve outcomes. It must be kept in mind that this technique is not suitable for all surgeons or all patients, but it results in excellent outcomes when applied correctly.

SVG occlusion risk prompts consideration of a second arterial graft or complete arterial revascularization. RITA and RA involve specific considerations, with similar long-term patency rates. Techniques like skeletonization harvesting and postoperative glycemic control can mitigate risks associated when using BITA in uncontrolled diabetes. For patients at high risk of redo sternotomy, techniques like covering the RITA graft with a Dacron graft or passing it through the transverse sinus may mitigate risks. The radial artery is preferred in uncontrolled diabetes and when its preservation is unnecessary for vascular access, such as in hemodialysis patients. Total arterial revascularization is advocated for maximizing long-term survival compared to single or double arterial grafting methods.

Hybrid revascularization remains an alternative for patients with significant LAD lesions and other suitable lesions for percutaneous revascularization. While clear recommendations are lacking, multidisciplinary decisions are suggested, offering advantages like shorter hospital stays and reduced costs.

## Data Availability

No datasets were generated or analysed during the current study.

## Abbreviations

ICM: Ischemic cardiomyopathy
LVD: Left ventricular dysfunction
LVEF: Left ventricular ejection fraction
CAD: Coronary artery disease
RCTs: Randomized controlled trials
CABG: Coronary artery bypass graft
PCI: Percutaneous coronary intervention
OMT: Optical medical therapy
CCS: Canadian Cardiovascular Society Angina Score
DSE: Dobutamine Stress Echocardiography
SPECT: Single Photon Emission Computed Tomography
CMR: Cardiovascular Magnetic Resonance
PET: Positron emission tomography
OPCABG: Off-pump coronary artery bypass grafting
EF: Ejection fraction
ONCABG: On-pump coronary artery bypass grafting
CPB: Cardiopulmonary bypass
IABP: Intra-aortic balloon pump
LAD: Left anterior descending artery
LITA: Left internal thoracic artery
HCR: Hybrid coronary revascularization
STS: Society of Thoracic Surgeons
ITA: Internal thoracic artery
MACE: Major adverse cardiovascular events
SVG: Saphenous vein graft
BITA: Bilateral internal thoracic artery
SITA: Single internal thoracic artery
RITA: Right internal thoracic artery
RCA: Right coronary artery
RA: Radial artery
DSWI: Deep sternal wound infections
HbA1c: Glycosilated hemoglobin
GEA: Gastroepiploic artery
IEA: Inferior epigastric artery
PAD: Peripheral artery disease
HFSA: Heart Failure Society of America
HFA/ESC: Heart Failure Association of the European Society of Cardiology
JHFS: Japanese Heart Failure Society
HFimpEF: Heart failure with improved ejection fraction
HFrEF: Heart failure with reduced ejection fraction
QoL: Quality of life
